# Histologic Evaluation of Human Pulp Response to Total Etch and Self Etch Adhesive Systems

**DOI:** 10.5812/ircmj.3335

**Published:** 2013-05-05

**Authors:** Mohammad Reza Malekipour, Sayed Mohammad Razavi, Saber Khazaei, Shantia Kazemi, Maryam Behnamanesh, Farzaneh Shirani

**Affiliations:** 1 Department of Operative Dentistry, Faculty of Dentistry, Azad University, Khorasgan (Isfahan Branch), IR Iran; 2 Torabinejad Dental Research Center and Department of Oral and maxillofacial Pathology, School of Dentistry, Isfahan University of Medical Sciences, Isfahan, IR Iran; 3 Dental Students’ Research Center, School of Dentistry, Isfahan University of Medical Sciences, Isfahan, IR Iran; 4 Faculty of Dentistry, Azad University, Khorasgan (Isfahan Branch), IR Iran; 5 Dental Materials Research Center and Department of Operative Dentistry, School of Dentistry, Isfahan University of Medical Sciences, Isfahan, IR Iran

**Keywords:** Pulp response, Single Bond, Prompt L-Pop, Adhesive system

## Abstract

**Background:**

To investigate pulp response to the application of two types adhesive systems (total-etch and self-etch) in human premolar teeth.

**Materials and Methods:**

Cavities limited to enamel walls in all margins with 2.5 mm depth were prepared on buccal surfaces of thirty three human premolars. The cavities were treated with the following adhesive. Single Bond (SB) and Prompt L-Pop (PLP). The teeth were extracted after 30 days and prepared due to histological technique.

**Results:**

Pulp responses were evaluated in three field including inflammatory cell response, pulp tissue disorganization and restorative dentin formation. There were no differences in histological response of the pulp tissue (P > 0.05).

**Conclusion:**

Both adhesive systems showed good biological compatibility.

## 1. Background

Inflammation of the pulp connective tissue like another defense mechanism is a damage to limit or prevent pulp inflammation (1). Bacteria play important roles in the pulp inflammation. It has been demonstrated that pulp damage without microbial contamination cannot be created (2).Inflammatory effects of dentin bonding materials were introduced in 1970 almost ten year before fusayama. Vojinovic et al. expressed that acid etched dentin increased permeability of the dentin (3, 4). White et al. describe the use of phosphoric acid on dentin and were permitted to use it (5). Akimoto et al., Ivanyi et al. and Medin et al. did not report the devastating effect of bonding systems on the pulp (6-8). It’s a necessity to know the effects and advers effects of different bonding systems on pulp, to achieve a sutiable performance of them. The aim of present study was to evaluate the histological response of pulp to the application of SB and PLP adhesive systems.

## 2. Materials and Methods

This study has been approved by Islamic Azad University, Khorasgan, Isfahan, Iran and has no conflict with Helsinki declaration. Thirty three human premolar teeth free of fillings, cervical abrasions, and caries that were scheduled to be removed for orthodontic purposes from eleven patients ranging in age from 12 to 20 years old. The tooth was radiographically examined to exclude presence of caries, cervical abrasions, or periapical pathologies. Electric pulp tester was applied to check the pulp vitality of all teeth. All teeth were polished with a rubber cup and prophylaxis paste at low speed and the surrounding field was cleaned with 70% isopropyl alcohol. Cavities were made on the cervical third of buccal surfaces of the clinical crowns of the premolars using a 012 straight-fissure diamond bur (D & Z, Diamate, Germany) in an air and water-cooled high-speed hand piece (2051). Cavities with the following dimensions (3.0 mm length, 2.5 mm depth, and 1.5 mm width) with margins limited to enamel were prepared on the buccal surfaces. Teeth were randomly assigned to three experimental groups, the control group and two other with different bonding agents (N = 11 for each group).

### 2.1. Group 1

Cavity walls were etched with 37% phosphoric acid gel (Scotchbond etchant;3M/ESPE, St Paul, MN) for 30 seconds and additional water was removed with a piece of absorbent paper 10 seconds later. SB (3M ESPE, Irvine, CA, USA) was used as the manufacturer’s instructions and then filled layered with composite Z100 (3M ESPE, A2 Shade, USA) in three layers and each layer was cured for 40 seconds at the last step.

### 2.2. Group 2

The entire process was carried out like the first group and the PLP system (3M ESPE, Sumaré, Brazil, Lot: 287452) was used and the cavity was filled with composite Z100. A light intensity light curing unit was used for resin polymerization in all samples (460 mW/cm ^2 ^) (Optilux 501, Kerr/Demetron, Danbury, CT, USA). Teeth were extracted 30 days after the intervention; teeth that didn’t received any intervention like control group were excluded. The extracted teeth were fixed in 10% formaldehyde, processed and finally hematoxylin-eosin staining was used to assess connective tissue reactions. After the above steps, glass slides got code number and were evaluated pathologically according to Table 1.

**Table 1.** Pulp Inflammatory Response

Score	Inflammatory cell infiltration	Soft tissue disorganization
**0**	None or few inflammatory cells in the pulp next to the axial surface or beneath the exposed dentinal tubules	Normal tissue morphology under the remaining dentin
**1**	Presence of acute or chronic inflammatory cells that most of them are PMNs (30>)	Odontoblastic disorganization below the remaining dentin with deeper pulp tissue appearing normal
**2**	Presence of many acute or chronic cells that most of them are MNs (30<)	Loss of general pulp morphology and cellular organization in the pulp
**3**	Severe inflammatory lesion appearing as an abscess or dense infiltrate involving at least one third of the pulp	Necrosis in at least coronal third of the pulp
**4**	Completely necrotic pulp	-

According to this table in three areas of pulp, cellular inflammatory response, soft tissue changes of pulp and restorative dentin formation were investigated.Mantel-Haenszel test was used for statistical analysis of pulp’s cellular inflammatory response and soft tissue changes of pulp between 3 groups and to compare the restorative dentin formation between 3 groups, Chi square test was done. P less than 0.05 were considered as significant level.

## 3. Results

**Figure 1.**
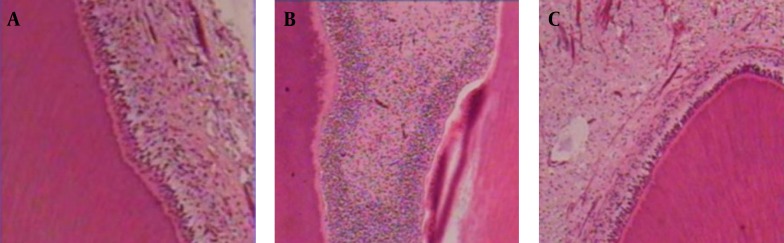
Control (A); PLP (B); SB (C) at 30 days. The pulp tissue in all groups is normal without inflammation. Magnification ×400; H and E stained

This study was done on eleven patients who need to extract at least three premolar teeth for orthodontic treatment and finally the statistical evaluation was carried out on forty six sections. Frequency of pulp’s cellular inflammatory response (P = 0.657) and degree of soft tissue changes in pulp (P = 0.321) between three groups showed no differences and also there were not any significant differences between the frequency of restorative dentin formation between three groups (Table 2) (Figure 1).

**Table 2.** Results of Pulp Response Severity in Different Groups

Inflammation	Control	Single Bond	Prompt L-Pop
**0**	3	2	1
**1**	4	5	4
**2**	0	0	2
**3**	0	0	0
**4**	0	0	0
**Pulp tissue disorganization**			
**0**	0	1	0
**1**	5	5	4
**2**	2	1	3
**3**	0	0	0
**Reparative dentin**			
**No**	5	5	3
**Yes**	2	2	4

## 4. Discussion

This study was done on human teeth, due to circumstances quite similar to common clinical approach of these materials. The results were closer to reality because of selecting a pair of teeth in one person which controls individual differences and changes in oral plaque. The time interval of 30 days after tooth extraction was need for maximum response to pulp inflammation. A time period of 30 days is required to compare the effects of restorative material and deposition of restorative dentin is often observed 28 days after stimulation (9). Cavities were limited to enamel walls in all margins and were not extended to dentinal edge for better bond of the composite resin and elimination of the microleakage which is known as the most potent factor for the pulp inflammation (10). Although the adhesions to the enamel in self etch system are not good enough in compare to the total etch system but because of aggressive reaction of self-etch system, the group in which PLP was applied, showed a stronger response and reaction in comparison to the control group, but this difference was not significant in none of the samples. Other studies reporting significant difference between PLP and SE/SB adhesive systems had prepared class V cavities with a gingival wall in the cervical margin which leads in inappropriate bond to dentinal wall and microleakge, respectively. But the present study had prepared cavities limited to enamel walls in all margins which results in elimination of the micro leakage and bacterial agents (10).Studies which prepared deep cavities showed significant difference between pulpal responses to current adhesive systems (11). But the current study had prepared low deep cavities and the residual dentinal thickness could buffer the adverse effect of the PLP on pulp. Even though the low number of specimens may be one limited factor of this study and significant difference may be expected by more number of specimens. According to many studies which suggest the microleakage as the most potent factor for pulpal inflammation in esthetic restorative materials, the present study did not found the adhesive agents irritant for the pulp in cavities with margins limited to enamel walls in the low depth used (12). There wasn’t any significant relation between the bonding agent type and grade of pulp inflammation. Lack of pulp inflammation in total etch adhesives can be explained by formation of a thick hybrid layer that can maintain normal tissue properties of dentin and prevent the creation of collagen without increased dentin permeability. Akimoto et al. investigated the clinical effects of two types of adhesive systems and Hebling et al. showed the application of adhesive systems with All-Bond 2 were similar to our study (6, 13). Tay et al. investigated the leverage influence of three self-etch adhesive systems and introduced PLP as the most potent group that can solve smear layer in all situations. PLP creates stable hybrid layer which is similar to the layer that created (14). Finally it can be noted that microbial leakage between the cavity walls and restoration is the main cause of pulp inflammation and sensitivity after treatment, so proper seal after cavity preparation will decrease Pulp and restoration sensitivity.
